# Editorial: Exploring evidence for neurorehabilitation advancements

**DOI:** 10.3389/fnhum.2025.1636154

**Published:** 2025-06-18

**Authors:** Hideki Nakano, Akiyoshi Matsugi, Tomotaka Ito, Kosuke Oku, Masahiro Sakita

**Affiliations:** ^1^Graduate School of Health Sciences, Kyoto Tachibana University, Kyoto, Japan; ^2^Faculty of Rehabilitation, Shijonawate Gakuen University, Daito, Japan; ^3^Faculty of Rehabilitation, Kawasaki University of Medical Welfare, Kurashiki, Japan

**Keywords:** rehabilitation, neuroscience, neural plasticity, brain function, motor recovery

## 1 Introduction

Neurorehabilitation is an important field dedicated to the restoration of function following nervous system injuries such as stroke or spinal cord injury. However, evidence regarding its efficacy and optimal approaches remains in the developmental stage, and many challenges persist. This Research Topic, titled “*Exploring evidence for neurorehabilitation advancements*,” compiles the latest research findings in the field of neurorehabilitation and provides a comprehensive examination of a wide range of themes including physical therapy, occupational therapy, neuromodulation technology, virtual reality, nutrition, and assessment methodologies. In this Editorial, we provide an overview of the key contributions of the 25 papers included in this Research Topic and discuss the latest advances in neurorehabilitation, their academic and clinical significance, and future challenges and prospects. By synthesizing the findings from each study, we aimed to clarify the current state of the neurorehabilitation field and offer guidance for future research and clinical applications.

## 2 Overview

This Research Topic includes a diverse array of contributions, from systematic reviews and meta-analyses to clinical trials, study protocols, and theoretical proposals, each focusing on a different aspect of neurorehabilitation.

First, this Research Topic contains several systematic reviews and meta-analyses evaluating the effects of rehabilitation interventions. One notable example is a meta-analysis that quantitatively examined the effects of physiotherapy on degenerative cerebellar ataxia (Matsugi et al.), which demonstrated significant improvements in gait ability and coordinated movement with physiotherapy interventions, supporting the utility of rehabilitation interventions for progressive diseases. In addition, a review by Lou et al. assessing the safety and efficacy of initiating very early rehabilitation in patients with acute stroke reported that early intervention may improve functional outcomes. Furthermore, Jiang et al. performed a comprehensive analysis of the effects of body-weight-supported treadmill training on balance and walking function in patients with stroke and found that rehabilitation interventions using assistive devices, such as body weight support systems, can contribute to improvements in balance and walking speed. However, a systematic review by Sánchez-González et al., which critically reevaluated the evidence for Vojta therapy, pointed out a lack of high-quality evidence regarding the clinical efficacy of the Vojta method for developmental and neurological disorders, highlighting the need for more rigorous validation. Additionally, a systematic review and meta-analysis of the effects of fully immersive virtual reality training in patients with mild cognitive impairment (MCI) showed that VR-based interventions led to significant improvements in the cognitive function of patients with MCI, supporting the effectiveness of rehabilitation approaches that leverage digital technology (Yu et al.).

This Research Topic also focused on non-invasive and invasive neuromodulation techniques. Regarding vagus nerve stimulation (VNS), an extensive literature review Korupolu et al. summarized strategies combining VNS with rehabilitation and reported its potential effects on motor recovery after stroke. In addition, a bibliometric study by Li et al., which analyzed research trends in transcranial magnetic stimulation (TMS) over the past 30 years, showed that the application of repetitive TMS (rTMS) for disorders such as aphasia and dysphagia has recently become a hot topic; however, further evidence is required for clinical implementation. Similarly, in another bibliometric analysis, Liu et al. examined global research trends in theta burst stimulation (TBS) and reported the recent increase in publications and major researcher networks. These reviews provide insights into the research trends for novel technologies and inform the planning of future research strategies.

This Research Topic also included a study that analyzes research trends in neurorehabilitation using bibliometric methods. Zhang and Zhu analyzed the literature trends in exercise therapy research for neurological diseases from 2000 to 2024 and reported a remarkable increase in the number of related publications and an expansion of collaborative relationships among major countries. Similarly, Hu et al. identified research hotspots in the field of microRNA studies for spinal cord injury, demonstrating that this field is gaining international attention for research on the molecular mechanisms of neural regeneration. Through these bibliometric analyses, the current global expansion of the evidence base in the field of neurorehabilitation and the directions of emerging research questions have been clarified.

Several original research studies have evaluated the effectiveness of novel rehabilitation approaches. For example, a randomized controlled trial by Sassmann et al. investigating electrical stimulation therapy for chemotherapy-induced peripheral neuropathy demonstrated a significant reduction in sensory impairment and pain, suggesting the potential of rehabilitation interventions to improve the quality of life of cancer survivors. Furthermore, in a primate model, Yan et al. implemented a novel approach aimed at upper limb functional recovery by applying epineural stimulation to the distal brachial plexus and showed that placing an electrode through a single axillary incision allowed selective activation of a broad range of muscles from the fingers to the upper arm. This finding suggests new possibilities for the application of invasive neuromodulation in the rehabilitation of severe paralysis. In another study, Sawai et al. compared the effects of different attentional foci (internal vs. external focus) on postural control in young and older adults and found that the predominant attentional focus significantly influenced standing balance in older adults, highlighting the importance of attentional focus in rehabilitation in this population. In a study by Valladares et al. investigating rehabilitation outcomes and related factors in stroke survivors, upper limb dexterity and motor impairment were assessed longitudinally, showing that improvements in fine motor skills either preceded or were strongly correlated with overall motor recovery. Furthermore, a prospective study by Rasová et al. observed that in patients with subacute stroke, intensive and comprehensive rehabilitation led to significant improvements in upper extremity muscle strength, dexterity, and independence in activities of daily living, with these effects particularly pronounced when intervention was initiated within a few weeks post-stroke.

Rehabilitation for COVID-19 sequelae has been addressed as a new challenge. Jöbges et al. evaluated the effects of inpatient rehabilitation in patients with post-COVID conditions (long COVID) (the PoCoRe study) and reported improvements in cognitive function and psychiatric symptoms, suggesting that comprehensive rehabilitation may be effective in alleviating long-term COVID symptoms. Demonstrating the role of rehabilitation in managing persistent symptoms of such emerging infectious diseases is important for meeting post-pandemic healthcare needs.

A review of the assessment methods for rehabilitation is also included. Wu and Jin conducted a systematic review of various scales for evaluating post-stroke fatigue in accordance with the COSMIN guidelines, comparing the reliability and validity of current measures and summarizing their strengths and weaknesses for clinical use. Similarly, a scoping review by Chen et al. on the impact of nutritional status on rehabilitation outcomes in patients with stroke indicated that patients with malnutrition tended to experience delayed functional recovery, underscoring the importance of nutritional management along with rehabilitation interventions. Furthermore, Guo et al. developed a novel nomogram to predict functional outcomes at 6 months post-intervention in patients with aneurysmal subarachnoid hemorrhage and reported high predictive accuracy. This prognostic tool is expected to provide clinicians with valuable information for early risk assessment and the planning of more effective rehabilitation strategies.

This Research Topic also included multiple protocol papers for future clinical trials, introducing efforts aimed at generating new evidence. For example, the study protocol by Wang et al., which outlines a large-scale trial using transcutaneous auricular vagus nerve stimulation (taVNS) in patients with disorders of consciousness, planned a multicenter double-blind randomized controlled trial with 382 participants and raised expectations for evaluating a novel treatment for severe disorders of consciousness. Additionally, Lee et al. presented a multicenter RCT protocol introducing personalized repetitive transcranial magnetic stimulation (rTMS) to enhance upper limb function in patients with stroke, an innovative attempt to verify whether an optimized stimulation protocol tailored to each patient can augment rehabilitation outcomes. Furthermore, a crossover trial protocol proposed by Xu et al. examined the effects of deep brain stimulation (DBS) in the mesencephalic locomotor region on gait function in patients with post-stroke hemiplegia, exploring the potential of a new therapy that combines surgical intervention with rehabilitation. Other protocols include a study by Xiao et al. that aimed to improve post-stroke upper limb paresis using a closed-loop taVNS device and a protocol by Reeder et al. that evaluated the implementation process of a comprehensive program integrating discharge planning and rehabilitation (the HOME Rehab program). These forward-looking studies on advanced rehabilitation techniques and their feasibility indicate that the field of neurorehabilitation is still in the process of building an evidence base and that deeper knowledge is being pursued through high-quality clinical research.

Finally, an intriguing study offered a novel theoretical proposal. Nakano et al. focused on the developmental mechanisms of interhemispheric interactions in the motor cortex of healthy individuals and proposed a theoretical model in which a new bimanual coordination training program leveraged these mechanisms to improve the paretic hand function. This study provides an innovative perspective by harnessing cerebral plasticity and developmental principles in stroke rehabilitation that may influence future research directions.

In summary, the 25 papers gathered on this Research Topic provide a broad spectrum of insights into neurorehabilitation, from basic science to applied clinical practice. Although each study addresses different target areas or intervention methods, they collectively contribute to the common goal of advancing evidence-based neurorehabilitation practices.

## 3 Recent advances in neurorehabilitation and their significance

Among the recent advances highlighted in relation to this Research Topic, improvements in both the quality and quantity of evidence are foremost. The growing collection of systematic reviews and meta-analyses integrated evidence of intervention effects not apparent in individual studies, thereby establishing a foundation for clinicians to select treatments based on scientific evidence (Jiang et al.; Matsugi et al.). For instance, providing quantitative evidence for the effectiveness of rehabilitation for cerebellar ataxia and the efficacy of body-weight–supported treadmill training offers clear support in areas that previously tended to rely on experience. In addition, progress has been made in elucidating the factors that influence rehabilitation outcomes, such as post-stroke fatigue and nutritional status (Wu and Jin; Chen et al.), thereby leading to renewed recognition of the importance of holistic approaches.

On the technological front, the application of neurotechnology in rehabilitation has become a prominent trend in recent years. Clinical studies on non-invasive brain stimulation (e.g., TMS/TBS) and vagus nerve stimulation (VNS) are increasing annually (Li et al.; Liu et al.), and multiple related papers have been published on this Research Topic. In particular, with regard to VNS, both a synthesis of existing studies (Korupolu et al.) and new efforts in device development and stimulation optimization (Xiao et al.) indicate that the use of neuromodulation technologies in rehabilitation is accelerating. Such cutting-edge technologies open new possibilities for addressing challenges such as severe paralysis and disorders of consciousness that were previously difficult to treat (Wang et al.; Xu et al.), and they serve to expand the horizons of neurorehabilitation.

Furthermore, the personalization and precision of rehabilitation medicine represents an important advancement. As seen in the protocol by Lee et al., the concept of individualized interventions, such as adjusting brain stimulation parameters for each patient, is emerging. This approach seeks to tailor rehabilitation strategies to each patient's specific brain network state rather than using one-size-fits-all interventions. It can potentially achieve greater efficacy and reduce side effects, thereby leading to more efficient training regimens.

Clinically, the effectiveness of comprehensive and intensive approaches has been reaffirmed (Rasová et al.; Jöbges et al.). Intensive rehabilitation by a multidisciplinary team and integrated programs during inpatient stays promote the recovery of upper limb function and activities of daily living and are beneficial even for patients with complex sequelae affecting cognitive, physical, and mental functions. These findings highlight the importance of rehabilitation delivery systems and support the appropriateness of early intensive interventions (Lou et al.).

Finally, bibliometric studies have underscored the global expansion of neurorehabilitation research and the importance of international collaborations (Hu et al.; Li et al.; Liu et al.; Zhang and Zhu). Analyses of highly productive countries and research networks provide a foundation for invigorating future international collaborations and information sharing. Moreover, tracking the evolution of hot topics can inform strategic planning to allocate research resources to address emerging questions (e.g., application of rTMS for aphasia or dysphagia).

As described above, the recent advances presented in papers on this Research Topic hold multifaceted significance. They include the enhancement of the scientific evidence base, introduction of cutting-edge technologies, personalization of treatments, value of comprehensive interventions, and promotion of research from an international perspective. Neurorehabilitation was once a field heavily reliant on experiential knowledge. However, a key message gleaned from this Research Topic is that it is steadily transitioning toward evidence-based practices.

## 4 Challenges and future research directions

The findings highlight remaining challenges. First, as noted in several review articles, high-quality evidence is lacking in several areas. For example, even if an intervention is reported to be effective, it is often supported by a limited number of RCTs or studies in small samples. A review addressing Vojta therapy demonstrated the fragility of its evidence base (Sánchez-González et al.), and further large-scale trials are required for the application of VNS in rehabilitation as well (Korupolu et al.). Therefore, it is necessary to reinforce these weak evidence areas through large multicenter trials and meta-analyses.

Second, in terms of technological development, the challenge lies in conducting clinical studies to verify the safety and efficacy of the new devices and stimulation methods. Promising concepts, such as closed-loop taVNS devices, have been proposed (Xiao et al.); however, whether they can improve clinical outcomes remains unclear. Similarly, although approaches such as personalized rTMS (Lee et al.) and novel DBS applications (Xu et al.) are intriguing, there are methodological challenges in the field of rehabilitation such as setting up placebo-controlled trials for device-based interventions. It is important to devise study designs that incorporate ethical and technical innovations to enable RCTs using cutting-edge methods.

Third, given that neurorehabilitation inherently addresses multifaceted functional recovery, implementing an integrated outcome assessment is challenging. In the PoCoRe study, a comprehensive evaluation, including cognitive and psychological aspects, was conducted (Jöbges et al.), but many studies have focused only on physical function metrics. Future research should enhance outcome assessment frameworks by including holistic outcomes such as patients' degree of community reintegration, quality of life, and psychological health (Wu and Jin). For example, it is important to take a multifaceted perspective on rehabilitation effects, examining, for instance, the extent to which improvements in dexterity or muscle strength ultimately increase independence in daily living, or whether they have ripple effects on cognitive function or motivation.

Furthermore, bridging the gap in clinical applications requires implementation research, i.e., research on methods to put evidence into practice. Even when an intervention is shown to be effective, there is insufficient insight on techniques to incorporate it into real-world rehabilitation programs in terms of training frequency and duration, staff education, and cost-effectiveness. It is hoped that more studies will include process evaluations of intervention implementation as seen in the HOME rehabilitation protocol in this Research Topic (Reeder et al.). As rehabilitation is highly context-dependent, comprehensive consideration, including environmental adjustments and organizational support, is required for translating research findings into practice.

Finally, human resource development and interdisciplinary collaboration are long-term issues. The utilization of cutting-edge technology and the promotion of personalized medicine require collaboration among rehabilitation professionals, engineers, neuroscientists, data scientists, and others (Li et al.; Liu et al.). Although studies on this Research Topic were conducted by multidisciplinary teams, further advancement through the establishment of interdisciplinary research centers and promotion of international collaborative projects is expected to accelerate innovation across the entire neurorehabilitation field.

## 5 Conclusion

The findings compiled in this Research Topic, titled “*Exploring evidence for neurorehabilitation advancements*,” demonstrate that the field of neurorehabilitation is steadily accumulating scientific evidence and forging new frontiers in both therapeutic technologies and strategies. Rehabilitation, which often relies on experience and intuition, is now transforming into an optimized approach for each patient based on precise assessment and evidence. The insights from each paper pertain to specific disorders or interventions in isolation; however, collectively, they form part of a larger movement toward the ultimate goal of neurorehabilitation: maximizing patients' functional recovery and improving their quality of life.

Certainly, many challenges must be overcome before the best possible rehabilitation, which is truly supported by evidence, can be realized. However, the types of efforts highlighted in this Research Topic, i.e., meticulous reviews to assess the current state, bold applications of new technology, intervention designs based on patient-centered thinking, and research pursued with an international outlook, will help resolving each of these challenges. Neurorehabilitation is a field supported by the hope afforded by the plasticity of the human brain and nervous system. To ensure concrete outcomes, continued collaboration between researchers and clinicians and the pursuit of innovative research are required.

The insights presented in this Research Topic offer valuable guidance for future research and clinical practice. By using these findings as a starting point and by continuously exploring new questions and accumulating evidence, neurorehabilitation will further develop. We are confident that this will ultimately lead to a future in which individuals with disabilities can fully realize their abilities and lead fulfilled lives. Through this Editorial, we hope that readers will, in their own capacities, contribute to the advancement of neurorehabilitation and share this vision for the future.

